# SLC10A4 regulates IgE-mediated mast cell degranulation in vitro and mast cell-mediated reactions in vivo

**DOI:** 10.1038/s41598-017-01121-8

**Published:** 2017-04-24

**Authors:** Hanna Pettersson, Behdad Zarnegar, Annika Westin, Viktor Persson, Christiane Peuckert, Jörgen Jonsson, Jenny Hallgren, Klas Kullander

**Affiliations:** 1 Department of Neuroscience, Uppsala University, Box 593, 751 24 Uppsala, Sweden; 2 Department of Medical Biochemistry and Microbiology, Uppsala University, Box 582, 751 23 Uppsala, Sweden; 3 Department of Organismal Biology, Uppsala University, Norbyv, 18A, 752 36 Uppsala, Sweden

## Abstract

Mast cells act as sensors in innate immunity and as effector cells in adaptive immune reactions. Here we demonstrate that SLC10A4, also referred to as the vesicular aminergic-associated transporter, VAAT, modifies mast cell degranulation. Strikingly, *Slc10a4* ^−/−^ bone marrow-derived mast cells (BMMCs) had a significant reduction in the release of granule-associated mediators in response to IgE/antigen-mediated activation, whereas the *in vitro* development of mast cells, the storage of the granule-associated enzyme mouse mast cell protease 6 (mMCP-6), and the release of prostaglandin D2 and IL-6 were normal. *Slc10a4*-deficient mice had a strongly reduced passive cutaneous anaphylaxis reaction and a less intense itching behaviour in response to the mast cell degranulator 48/80. Live imaging of the IgE/antigen-mediated activation showed decreased degranulation and that ATP was retained to a higher degree in mast cell granules lacking SLC10A4. Furthermore, ATP was reduced by two thirds in *Slc10a4* ^−/−^ BMMCs supernatants in response to IgE/antigen. We speculate that SLC10A4 affects the amount of granule-associated ATP upon IgE/antigen-induced mast cell activation, which affect the release of granule-associated mast cell mediators. In summary, SLC10A4 acts as a regulator of degranulation *in vitro* and of mast cell-related reactions *in vivo*.

## Introduction

Mast cells are immune cells which play a central role in allergic reactions^1^. The most well-characterized pathway of mast cell activation is the IgE/antigen-mediated activation of the high-affinity receptor for IgE, FcεRI. This mode of activation triggers a complex sequence of reactions inside the mast cells that lead to an immediate Ca^2+^- dependent exocytosis of granular mediators into the extracellular space. In addition, IgE/antigen-mediated activation stimulates production and release of lipid mediators, i.e. prostaglandins, leukotrienes and a vast array of cytokines^2^. Mast cell granules contain mast cell-specific proteases: tryptase, chymase and carboxypeptidase A, which are presumably sorted into the mast cell progranules directly from the trans-golgi network or gradually during granule maturation^3^. However, for many of these proteases, including the mouse tryptase mMCP-6, retention in the granules is dependent on the presence of negatively charged heparin/chondroitin sulphate E proteoglycans^4, 5^. In the case of mMCP-6, positively charged histidine residues exposed on the surface of the active tetramer are likely to mediate the interaction with these proteoglycans^6^. Mast cell granules also contain mediators that are shared by other cells such as histamine, serotonin, ATP and lysosomal enzymes such as β-hexosaminidase^3^. In contrast to the mast cell proteases, the biogenic amines such as histamine enter the granules through the vesicular monoamine transporter 2 (VMAT2)^7^.

In similarity to mast cells, neurons release mediators in a regulated fashion even if their time frame of exocytosis and recycling is considerably shorter^2^. Some of the exocytosed mediators are common to mast cells and neurons, *e.g*. histamine, which binds and activates G protein-coupled histamine receptors (H1–H4), and serotonin, another monoamine neurotransmitter, which signals through an array of different G-protein coupled 5-HT-receptors^8, 9^. These mediators play essential roles in the inflammatory responses and as neurotransmitters in the nervous system.

The *Slc10a4* gene encodes SLC10A4 also referred to as the vesicular aminergic-associated transporter, VAAT, which has primarily been associated to functionality of the aminergic systems^10^. SLC10A4 was originally identified as an expressed sequence tag and was designated, based on sequence similarity, as a novel member of the solute carrier family 10 (SLC10) family^11^. The protein was cloned and characterized by Splinter and co-workers in 2006^12^. SLC10 is known as the sodium bile acid co-transporter family, since its seminal members, SLC10A1 (NTCP) and SLC10A2 (ASBT), are the bile acid transporters of the liver and the gut, respectively^13–15^. In spite of significant efforts, the substrate(s) of SLC10A4 still essentially remains unknown^10, 12, 16–18^. Two studies have established that SLC10A4 is co-expressed with the carriers of acetylcholine (VAChT) and monoamines (VMAT2) on synaptic vesicles, both in the central and peripheral nervous systems^10, 16^. This suggested the presence of SLC10A4 in other monoamine-containing secretory granules, which was supported by the identification of the SLC10A4 protein in rat peritoneal mast cells^19^. While a role for SLC10A4 in the dopaminergic and cholinergic systems has been established^10, 20^, its role in mast cells has so far been unknown. In this study, we show that the SLC10A4 protein impacts the degranulation process of mast cells *in vitro* and regulates mast cell-mediated responses *in vivo*.

## Results

**Figure 1:**
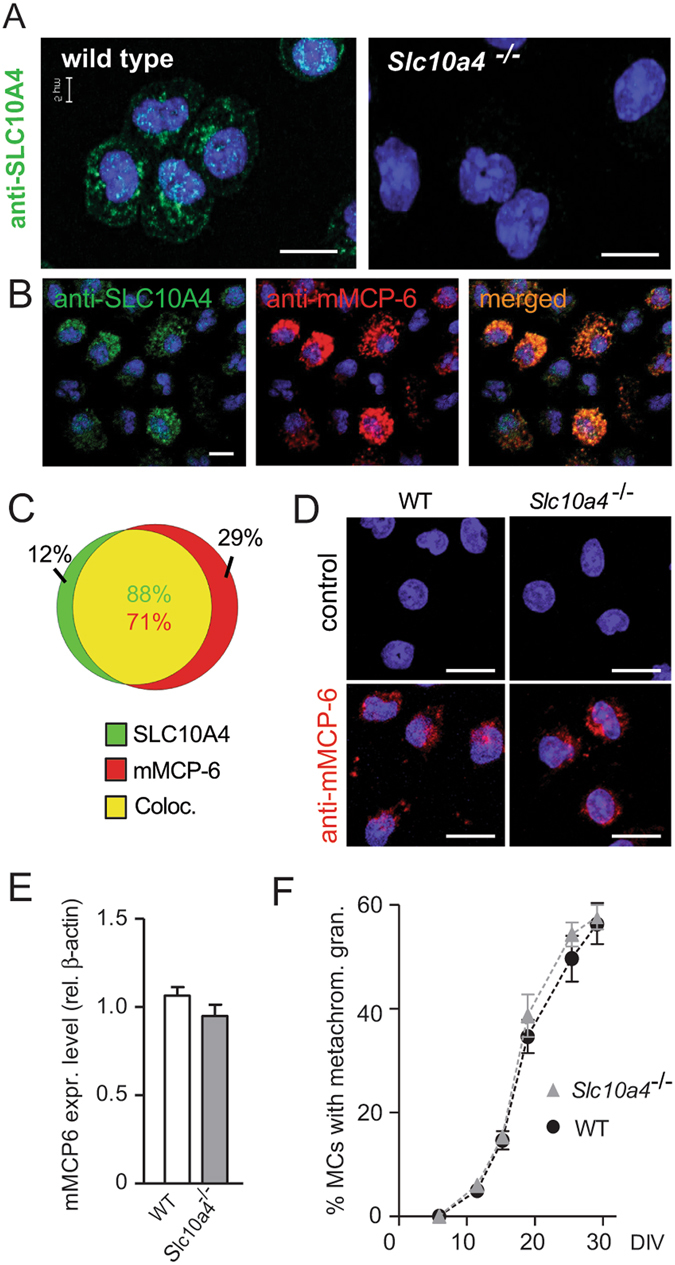
SLC10A4 co-localises with mMCP-6 and lack of SLC10A4 does not interfere with the storage of mMCP-6 inside the mast cell granules. (**A**) Wild type and *Slc10a4* ^−/−^ BMMCs were immunostained with anti-SLC10A4 antibody (green), counterstained with DAPI (blue) and analysed by immunofluorescence microscopy. (**B**) Wild type BMMCs were stained with anti-SLC10A4 (green) and anti-mMCP-6 (red) antibodies and counterstained with DAPI to visualize granular staining. (**B**,**C**) The majority of the mast cell granules demonstrated co-localization (yellow) of mMCP-6 and SLC10A4 as indicated in the Venn diagram (analysis of 112 wild type BMMCs). (**D**) Representative pictures of wild type (WT) and *Slc10a4* ^−/−^ BMMCs immunostained for mMCP-6 (red) and counterstained with DAPI (blue). As a negative control, BMMCs were stained with the secondary anti-goat antibody alone (control). (**E**) Quantitative western blot analysis of mMCP-6 expression in BMMCs derived from WT and *Slc10a4* ^−/−^ mice. The quantification analyses were performed by western blot on cell lysate and mMCP-6 expression levels were normalised to the β-actin amount in the same sample. The data shown are presented as mean ± SEM from six samples obtained from three independent western blots. No significant difference (p = 0.18) was found using a standard unpaired Student’s t-test. (**F**) WT and *Slc10a4* ^−/−^ bone marrow cells were cultured with SCF and IL-3 to obtain BMMCs. Samples from the indicated days after the start of the cultures *in vitro* (days *in vitro*; DIV) were cytospun, stained with May-Grünwald/Giemsa and evaluated as positive or negative for cells with metachromatic staining. These data represent pooled data from two mice per genotype where the percentage of cells with metachromatic staining were calculated from the individual cell cultures based on three individual cytospins per data point. Scale bars, 10 µm.

We first set out to determine whether SLC10A4 was expressed by mouse mast cells. Bone marrow cells from *Slc10a4*-deficient and wild type mice were cultured in the presence of IL-3 and stem cell factor (SCF) to obtain c-kit^+^ FcεRI^+^ mast cells. Immunohistochemistry analyses demonstrated that SLC10A4 was expressed in BMMCs from wild type mice whereas the *Slc10a4* ^−/−^ BMMCs lacked staining of SLC10A4 protein (Fig. 1A). SLC10A4-specific immunoreactivity was previously demonstrated in secretory granules of rat peritoneal mast cells using immunohistochemistry and electron microscopy and found to be partly co-localized with VMAT2^19^. In western blot experiments, SLC10A4 protein was enriched together with the synaptic vesicle protein synaptophysin throughout the purification steps of a rat brain vesicle preparation. Moreover, analysis of purified synaptic vesicles enriched from wildtype and knockout mice, confirmed expression of SLC10A4 localized to synaptic vesicles and absence of SLC10A4 in *Slc10a4* ^−/−^ mice^10^.

### SLC10A4 co-localises with mMCP-6 and the lack of SLC10A4 does not interfere with the storage of mMCP-6 in the mast cell granules

To further investigate the localisation of SLC10A4 protein in mast cells, wild type BMMCs were co-stained with antibodies toward SLC10A4 and the mast cell specific and granule localized protease, mMCP-6, and analysed by immunofluorescence microscopy. SLC10A4 immunopositive signals were found to overlap with mMCP-6 staining signals, suggesting an association of SLC10A4 with mouse mast cell granules (Fig. 1B). Quantification based on fluorescence signals showed that 88% of the fluorescence from SLC10A4 positively-stained granules overlapped with fluorescence from mMCP-6 positively stained granules. Conversely, 71% of the fluorescence from mMCP-6 positive granules overlapped with SLC10A4 positively-stained granules (Fig. 1C). Moreover, immune staining of mMCP-6 in wild type (age- and sex-matched littermate controls) and *Slc10a4* ^−/−^ mast cells suggested that the storage of mMCP-6 protein in the granules was intact in mast cells lacking SLC10A4 (Fig. 1D). Western blot analysis of mMCP-6 protein levels confirmed this observation (Fig. 1E).

To examine whether the lack of SLC10A4 was interfering with normal *in vitro* development of mast cells in the bone marrow cultures, wild type and *Slc10a4* ^−/−^ bone marrow cells were cultured in parallel and the maturation of mast cells was monitored. Cell samples taken from the cultures in regular intervals during the differentiation were stained with May-Grünwald/Giemsa and evaluated for their content of metachromatic mast cell granules. Overall, there were no differences in the staining properties or maturation into mast cells between wild type and *Slc10a4* ^−/−^ BMMCs (Fig. 1F). In addition, the BMMC cultures were routinely analysed by flow cytometry for c-kit and FcεRI expression before further experimentation. In these analyses, there were no differences in the proportion of c-kit^+^ FcεRI^+^ mast cells after three-four weeks of culture (Supplementary Fig. S1). Furthermore, a similar percentage of c-kit^+^ FcεRI^+^ peritoneal mast cells was observed in wild type and *Slc10a4* ^−/−^ mice using flow cytometry (Supplementary Fig. S2). In summary, SLC10A4 was found localized to the mast cell granules and lack of SLC10A4 did not interfere with the normal maturation process *in vitro* or the storage of mMCP-6 in the mast cell granules.

### SLC10A4 is required for optimal IgE-mediated mast cell degranulation

**Figure 2:**
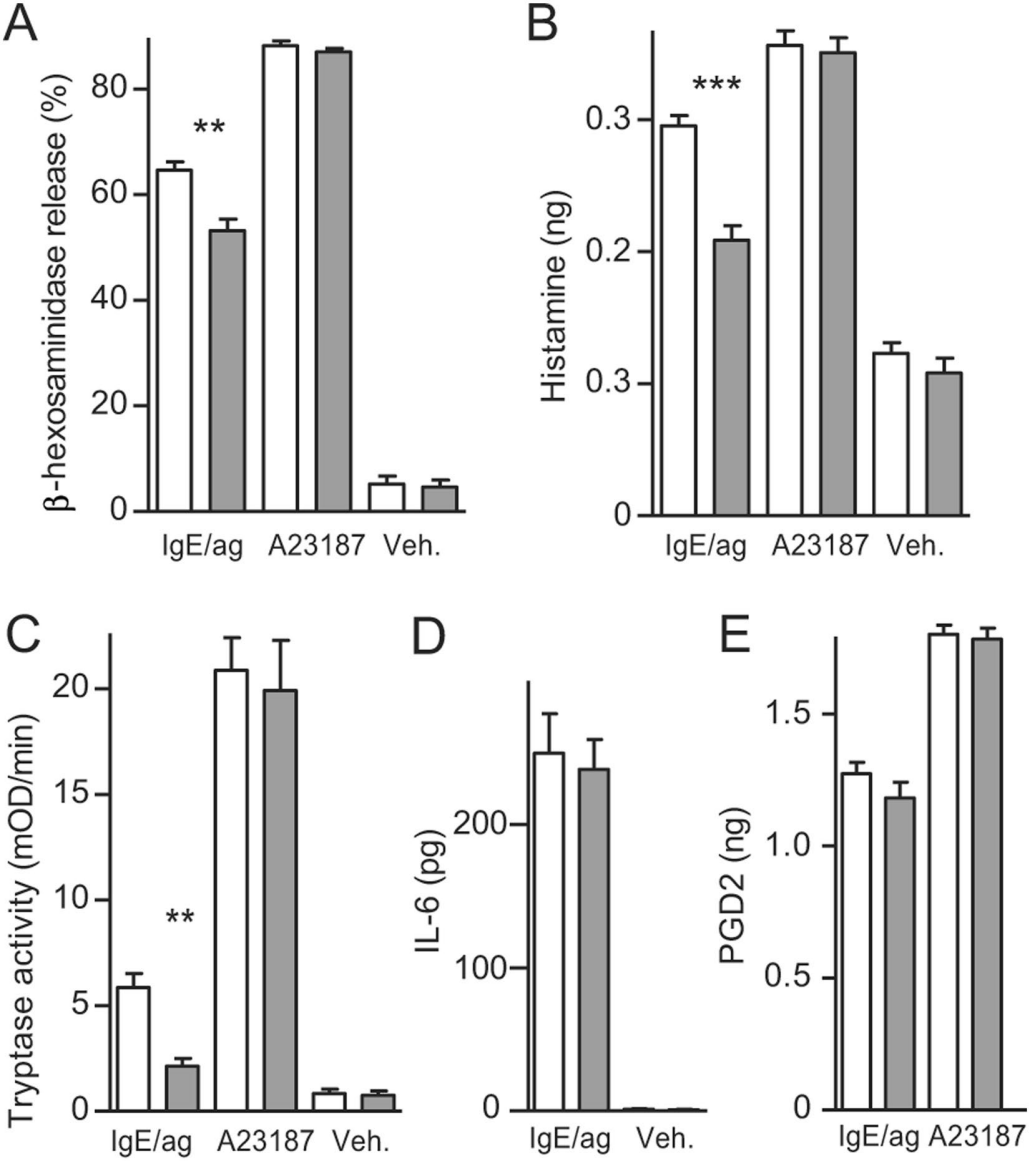
Reduced release of granule-associated mediators from mast cells lacking SLC10A4 after IgE/antigen-induced activation. (**A–E**) Wild type (white) and *Slc10a4* ^−/−^ (grey) BMMCs were sensitized with IgE anti-TNP and treated with either OVA-TNP (IgE/ag), Ca^2+^-ionophore (A23187) or vehicle alone (Veh.). The data were obtained from three individual BMMC cultures from two mice per group, each treatment was performed in triplicates and analysed in quadruplicates. The data shown are presented as mean ± SEM. (**A**) β-hexosaminidase in the cell fraction and supernatants were analysed after one hour activation and the percent release calculated. (**B**) The amount of histamine from a million cells released into the supernatants. The data shown in (**A,B**) are from one representative experiment out of three. (**C**) Tryptase activity in the supernatants was analysed after one hour incubation. The data shown are from one representative experiment out of five. (**D**) The amount of released IL-6 from a million cells was quantified by ELISA after 18–24 hours of incubation. The data shown are from one representative experiment out of four. (**E**) The amount of released prostaglandin D2 (PGD2) from a million cells was quantified by ELISA after one hour incubation. The data shown are from one representative experiment out of three. Asterisks mark significant differences (ns, non-significant, **P < 0.01, ***P < 0.001), Student’s t-test.

We next tested whether SLC10A4 is involved in IgE/antigen-mediated mast cell degranulation, BMMCs were sensitized with anti-2, 4, 6-trinitrophenyl (TNP) IgE and challenged with ovalbumin conjugated to TNP (OVA-TNP) as a model antigen. The Ca^2+^-ionophore, A23187, and vehicle were included as positive and negative controls, respectively. First, β-hexosaminidase was quantified in the supernatant and in the cellular fraction of the BMMCs. Application of the Ca^2+^-ionophore stimulated the release of around 90% of the granular content of β-hexosaminidase in both wild type and *Slc10a4* ^−/−^ BMMCs (Fig. 2A). OVA-TNP stimulation of sensitized wild type BMMCs resulted in approximately 65% release of β-hexosaminidase whereas such IgE/antigen-mediated release of β-hexosaminidase from the *Slc10a4* ^−/−^ BMMCs was significantly reduced (Fig. 2A). Moreover, the histamine levels and tryptase activity were significantly reduced in the supernatants of *Slc10a4* ^−/−^ BMMCs after IgE/antigen stimulation compared with the wild type control BMMCs (Fig. 2B,C). Nevertheless, Ca^2+^-ionophore treatment, which causes unregulated release of virtually all granules, resulted in similar release of these granule-associated mediators in wild type and *Slc10a4* ^−/−^ BMMCs (Fig. 2B,C). We next set out to determine whether the SLC10A4-mediated reduction in mediators released after IgE/antigen stimulation was specific for granule-associated mediators or had a global effect on mediator release, and therefore quantified IL-6 and prostaglandin D2 levels in the supernatants after stimulation. Both IL-6 and prostaglandin D2 were present at similar levels in the supernatants from wild type and *Slc10a4* ^−/−^ BMMCs after IgE/antigen-mediated activation (Fig. 2D,E). Together, these data suggest that SLC10A4 is involved in mast cell degranulation but not in the release of cytokines and lipid mediators after IgE/antigen-mediated activation.

### Mast cells lacking SLC10A4 release less ATP in response to IgE-mediated degranulation

ATP is a granular component that may promote vesicular filling of monoamines, possibly by counterbalancing these positively charged molecules by the negatively charged ATP^10^. To study whether the kinetic of the IgE/antigen-mediated degranulation process was modified in mast cells lacking SLC10A4, we next performed live cell imaging of cellular ATP. Briefly, BMMCs were stained with quinacrine, an acridine derivative known to bind ATP^21, 22^, before they were subjected to IgE/antigen-mediated activation. It has been demonstrated that the release of secretory granule content is associated with a decrease in quinacrine fluorescence in several different cell types^23–25^. The quinacrine staining, which appeared granular in the raw images, was processed by the Imaris software from point type signals to spheres in order to calculate the volume. The fluorescent signals resulted in imaged and quantifiable volumes of the mast cells, which presumably reflects intragranular ATP levels.

**Figure 3:**
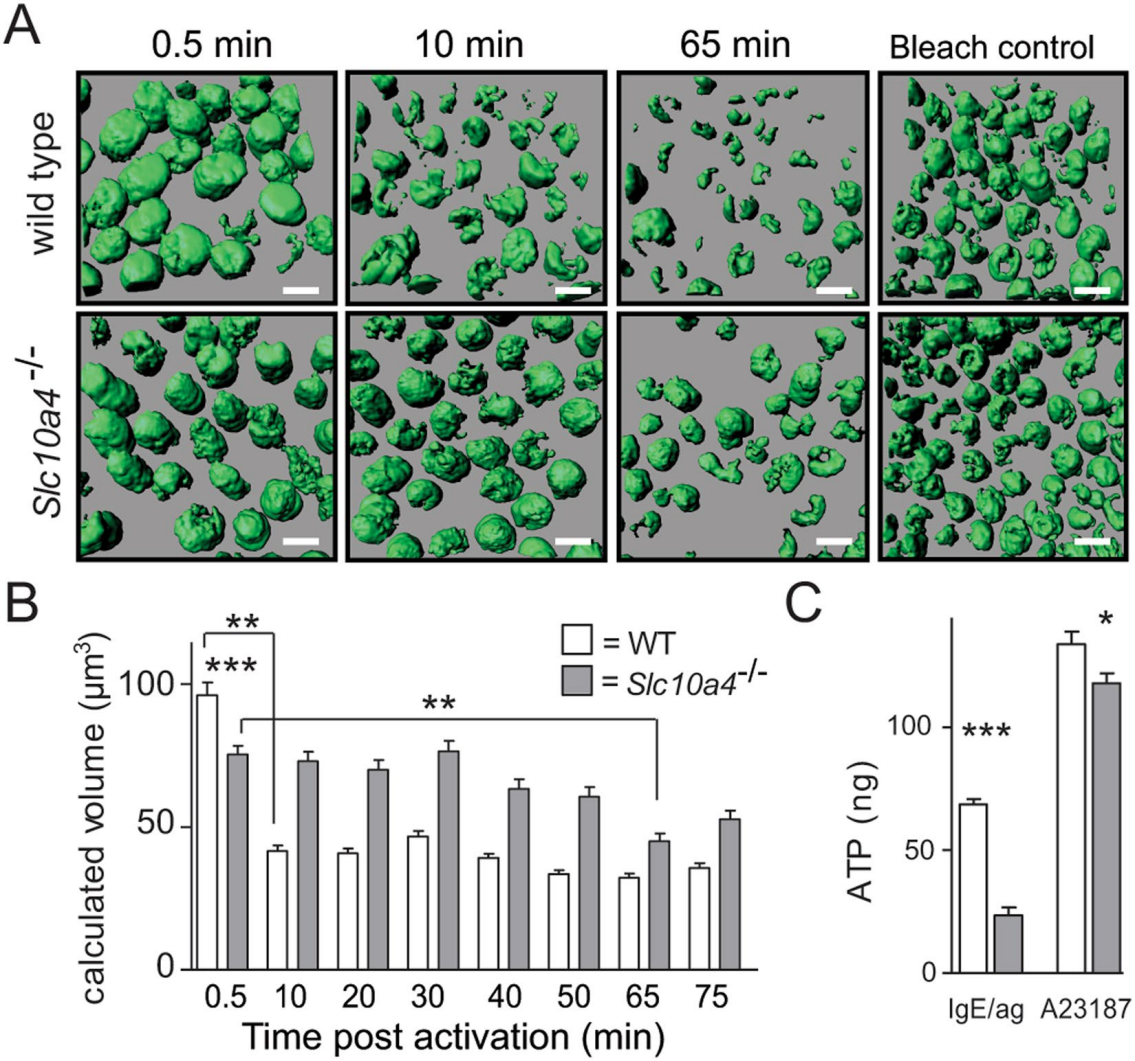
Mast cells lacking SLC10A4 release less ATP in response to IgE/antigen-mediated activation and stimulation with a Ca^2+^-ionophor. (**A**,**B**) Anti-TNP IgE-sensitized wild type and *Slc10a4* ^−/−^ BMMCs were stained with the ATP-binding drug quinacrine (green) and treated with OVA-TNP to trigger degranulation. The reactions were monitored over 75 min. As a control, untreated BMMCs were left in the confocal microscope for monitoring the decrease in signal due to the laser exposure for the same time span (Bleach control). Bar = 10 µm. (**A**) Representative pictures from one experiment out of two from the live cell time-lapse microscopy at 0.5, 10 and 65 min after stimulation. (**B**) Quantification of the granular fluorescent signals transformed into volume after IgE/antigen-mediated activation. The data shown are the mean ± SEM from two pooled independent experiment showing in total 600 *Slc10a4* ^+/+^ and 520 *Slc10a4* ^−/−^ BMMCs. Asterisks mark the most interesting significant differences (**P < 0.01, ***P < 0.001) obtained with Kruskal-Wallis test followed by Dunn’s multiple comparison post-hoc analysis. A full summary of all statistical comparisons is shown in Table 1. (**C**) Wild type (WT) and *Slc10a4* ^−/−^ BMMCs were sensitized with anti-TNP IgE and subsequently challenged with OVA-TNP (IgE/ag) or given Ca^2+^-ionophore (A23187). After one hour, the cell supernatants were analysed for ATP content using a Luciferin-Luciferase Bioluminescence assay. The data shown are the mean ± SEM from three independent experiments. Asterisks mark significant differences (*P < 0.05, ***P < 0.001) performed using Student’s t-test.

Sensitized wild type and *Slc10a4* ^−/−^ BMMCs determined by the cellular fluorescent signals show similar ATP levels before activation (Supplementary Fig. S3). The fluorescent signals from wild type BMMCs were significantly reduced during the first 10 minutes after IgE/antigen-mediated mast cell activation as a result of a significant decrease in the calculated volume of the wild type mast cells after degranulation (Fig. 3A,B). The fluorescent signal of the wild type BMMCs remained at a similar level from 10 to 75 minutes post-activation, suggesting that the wild type mast cells were fully degranulated already after 10 minutes post-activation. In contrast, the *Slc10a4* ^−/−^ BMMCs had smaller calculated volume than wild type BMMCs immediately after IgE/antigen stimulation (t ∼0.5 min) (Fig. 3A,B and Table 1). Further, the cellular volume of *Slc10a4* ^−/−^ BMMCs remained at the approximately same level from 0.5 min until 65 min post-activation when there was a significant decrease in fluorescent signal compared to 0.5 min (Fig. 3A,B and Table 1). These results suggested that *Slc10a4* ^−/−^ mast cells, after a quick partial degranulation, degranulated very slowly. Furthermore, between 10–75 minutes post-activation, the cellular volume of wild type BMMC remained smaller than the *Slc10a4* ^−/−^ BMMCs (Table 1), suggesting that mast cells lacking SLC10A4 never reached the same level of degranulation as their wild type counter parts.

**Table 1:** Complete statistical analysis of the data presented in Fig. 3B.

Time (min)	WT vs KO	Comparisons	KO vs KO at 0.5 min
		WT vs WT at 0.5 min	
0.5	***	—	—
10	***	**	ns
20	***	**	ns
30	***	**	ns
40	***	**	ns
50	***	**	ns
65	***	**	**
75	***	**	ns

Wild type (WT) and *Slc10a4* ^−/−^ (KO) BMMCs were compared to each other at each time point, and the volume at time point 0.5 min was compared to the later time points. Asterisks mark significant differences obtained with Kruskal-Wallis test followed by Dunn’s multiple comparison post-hoc analysis (ns, non-significant, **P < 0.01, ***P < 0.001).

We further quantified ATP in the supernatants that were analysed for mast cell mediators (depicted in Fig. 2) by a Luciferin-Luciferase bioluminescence assay one hour after IgE/antigen-mediated activation. Around three times more ATP was detected in the wild type BMMC supernatants compared with the supernatants from *Slc10a4* ^−/−^ BMMCs (Fig. 3C). This indicates that release of ATP upon IgE/antigen-mediated degranulation was strongly reduced in *Slc10a4* ^−/−^ mast cells. In addition, there was also less ATP detected in the supernatants from the *Slc10a4* ^−/−^ BMMCs after stimulation with a Ca^2+^-ionophore (Fig. 3C), suggesting that SLC10A4-deficient mast cells stored less granular ATP. These data suggest that loss of SLC10A4 led to a decreased ATP availability in mast cell granules, which resulted in a decreased release of granular ATP upon IgE/antigen-mediated mast cell activation.

### Mice lacking SLC10A4 demonstrate reduced passive cutaneous anaphylaxis reactions

**Figure 4:**
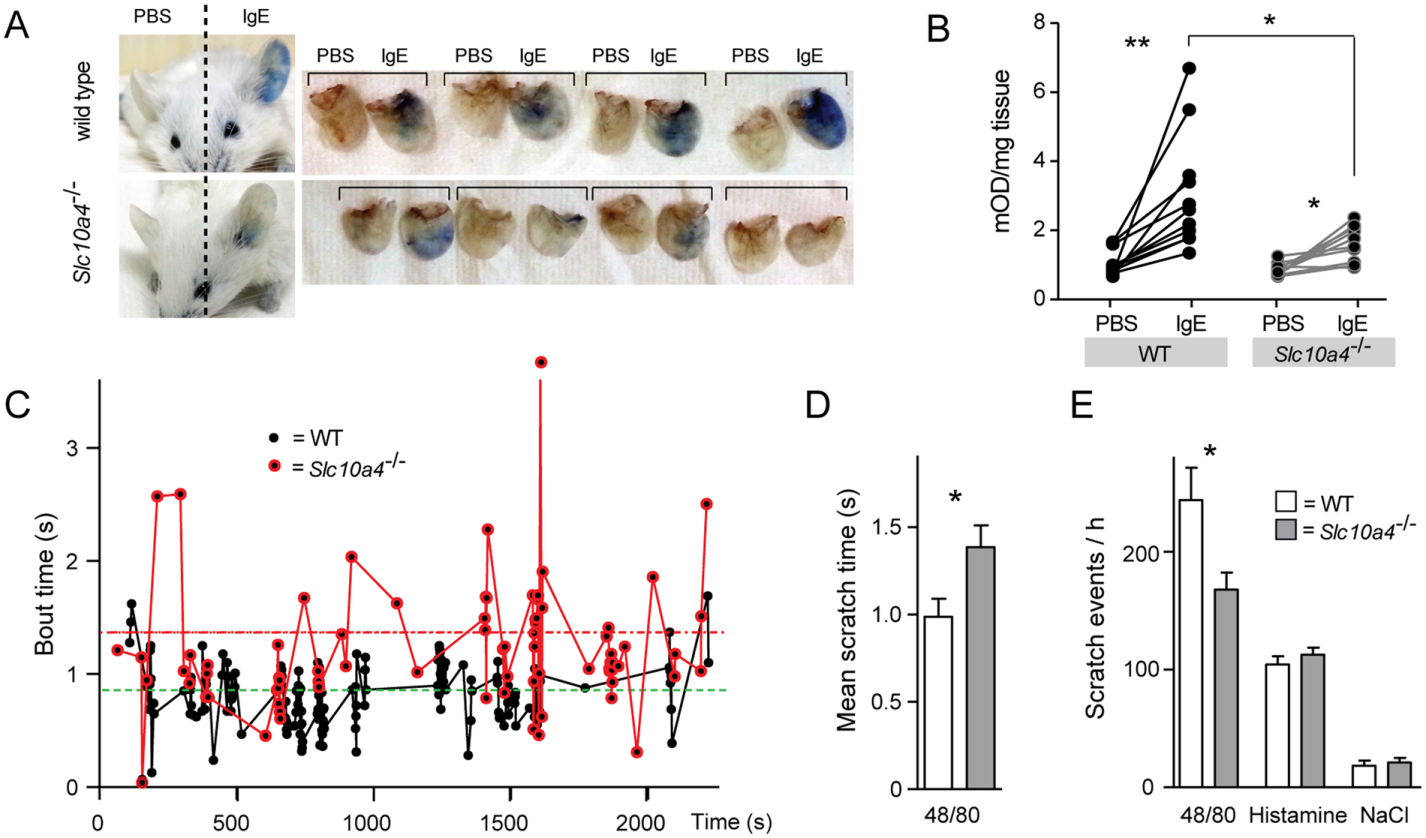
Mice lacking SLC10A4 demonstrates decreased mast cell-mediated responses *in vivo*. (**A,B**) Wild type (WT) BALB/c and *Slc10a4* ^−/−^ littermates were sensitized by intradermal injection of anti-OVA IgE (left ear) and PBS (right ear). The following day, all mice were given OVA in Evans Blue dye intravenously in the tail. (**A**) Representative pictures of WT and *Slc10a4* ^−/−^ mice and ears. (**B**) The quantifications of extravasated Evans Blue dye per mg tissue from 10–11 individual mice per group from a representative experiment out of two. Asterisks mark significant differences (*P < 0.05, **P < 0.01) using a paired Student’s t-test. (**C–E**) WT and *Slc10a4* ^−/−^ mice were given intradermal neck injections of substance 48/80, histamine or saline (NaCl) as vehicle and their scratching behaviour was recorded during 60 min and analysed for the number of scratching events and the duration of each scratching event (bout time). (**C**) The bout time pattern from a representative WT and *Slc10a4* ^−/−^ mouse after activation by 48/80. Each dot represents a scratching event. (**D,E**) The data shown are the mean ± SEM of 10 mice per group genotype and treatment (48/80, histamine or NaCl) from a single experiment. Asterisks mark significant differences (*P < 0.05) using a standard unpaired Student’s t-test.

To determine if the decreased release of granule-associated mediators in mast cells lacking SLC10A4 would also have an effect on mast cell-related reactions *in vivo*, we turned to the IgE-mediated passive cutaneous anaphylaxis (PCA) model. This model evaluates mast cell-mediated allergic reactions^26–28^, and more specifically, the immediate dermal response to mast cell activation that leads to release of mediators with increased permeability of vessels within the skin as a result. The response is visualized and quantified by Evans blue, which binds to and is extravasated with plasma albumin. To readily visualize the blue PCA reaction in less pigmented skin, *Slc10a4* ^−/−^ mice were backcrossed into the BALB/c background. Wild type control littermates and *Slc10a4* ^−/−^ mice were sensitized with anti-OVA IgE in the left ear and PBS in the right ear. Subsequent intravenous injection of OVA/Evans Blue in wild type controls resulted in a blue colour in the ears injected with anti-OVA IgE, but not in the PBS-injected ears (Fig. 4A,B). In contrast, *Slc10a4* ^−/−^ mice showed significantly attenuated blue colour extravasation in the anti-OVA IgE-injected ears after OVA/Evans Blue injection when compared to wild type controls (Fig. 4A,B). These data suggest that loss of SLC10A4 affected the release of histamine from mast cells *in vivo*.

### Mice lacking SLC10A4 have a decreased scratching frequency after 48/80 injection

Within the epidermis, free nerve endings of non-myelinated C-type nerves mediate itch. A sub-population of these non-myelinated C nerve fibers respond specifically to histamine, predominantly through histamine H1 receptors but also through H4 receptors^29^. Since mast cells reside in close proximity to blood vessel and nerves in the skin and store preformed histamine within their granules, mast cells are considered to play a central role in histamine-mediated itch reactions. We sought to determine whether SLC10A4 might play a role in mast cell-mediated reactions independent of the IgE/antigen activation pathway *in vivo*. For this purpose, wild type and *Slc10a4* ^−/−^ mice, were given the mast cell degranulation compound 48/80 intradermally in the neck, as well as histamine or vehicle (saline) as positive and negative controls, respectively. Compound 48/80 induces mast cell degranulation through interaction with the G-protein coupled receptor Mrgprb2^30^. All 48/80- or histamine-injected mice responded with increased scratching behaviour whereas vehicle injections did not induce an increased scratching behaviour, regardless of genotype (Fig. 4E). However, the pattern of scratching frequency and duration was different between the wild type and *Slc10a4* ^−/−^ mice upon 48/80 injections. While *Slc10a4* ^−/−^ mice in average had longer accumulated scratching time compared to wild type mice (Fig. 4C,D), *Slc10a4* ^−/−^ mice displayed significantly fewer scratch events than wild type controls (Fig. 4E). In contrast, histamine injections produced similar scratching responses in wild type and *Slc10a4* ^−/−^ mice, *i.e*. they displayed similar average scratching times (results not shown) and number of scratch events (Fig. 4E). Altogether, these data implicate SLC10A4 in 48/80-mediated scratch reactions.

## Discussion

Cross-linking of IgE bound to the FcεRI by antigen causes strong mast cell activation, which results in the degranulation of mast cells and in the generation of lipid mediators and cytokines. Here, SLC10A4 in BMMCs was co-localized with a granule-associated protease and the loss of SLC10A4 in these cells led to a reduced release of granule-associated mediators after IgE/antigen-mediated activation. This implicates a role of SLC10A4 in the mast cell granules, which has a functional consequence during IgE/antigen-mediated degranulation.

The intracellular cascade of events that takes place in mast cells after IgE/antigen-mediated activation of FcεRI results in activation of src family kinases Lyn and Fyn and in the phosphorylation of downstream kinases, which phosphorylates immunoreceptor tyrosine-based motifs (ITAMs) on the β- and γ-chains of FcεRI^31^. These events trigger calcium channels to open in the plasma membrane causing a receptor-operated calcium entry (ROCE)^2, 32^ that eventually lead to the activation of the IP_3_ receptor on the endoplasmatic reticulum (ER), which stimulates Ca^2+^ release from ER. This induces a conformational change in STIM1^33^, which then translocates to specific junctions between ER and the plasma membrane and interacts with Orai1 and other calcium channel proteins to form Ca^2+^ release-activated Ca^2+^ (CRAC) channels^34, 35^. Calcium enters from the extracellular environment through such channels^35^ and the increases in intracellular Ca^2+^ regulate the release of lipid mediators in mast cells^36^. SLC10A4-lacking mast cells were able to release normal levels of prostaglandin D2 in response to IgE/antigen-mediated activation, suggesting that calcium channel formation was normal. Furthermore, the normal IL-6 release after IgE/antigen-mediated cross-linking also suggests that the signalling pathways that regulates cytokine production and release were also intact in SLC10A4-deficient mast cells.

The increase in intracellular Ca^2+^ levels can be accomplished externally by the addition of Ca^2+^-ionophores like A23187, which was used as a positive degranulation control in this study. A23187 facilitates the increase in intracellular Ca^2+^ levels by a non-receptor mediated mechanism through redistribution of intracellular Ca^2+^ pools into the cytosol^37, 38^, which trigger degranulation through activation of protein kinase C (PKC)^39, 40^. *Slc10a4* ^−/−^ BMMCs showed a reduced release in granule mediators in response to IgE/antigen-mediated activation of FcεRI, whereas the release of granule mediators in response to Ca^2+^-ionophore was essentially normal. This suggests that receptor-mediated signalling events were required to detect the influence of SLC10A4 on granule mediator release. Likely, the FcεRI-mediated mast cell activation cascade that ends with granule fusion is highly regulated, which is in contrast to the general activation by Ca^2+^-ionophores such as A23187. Exactly how the early signalling events leading to Ca^2+^ influx and the late signalling events monitoring exocytosis of granule components are coupled is unknown^2^. The degranulation process, including exocytosis of granule-associated mediators, is regulated by SNAREs and is ATP-dependent^41, 42^. SNAREs, vesicle-associated membrane proteins involved in the docking and/or fusion of vesicles with the plasma membrane, have a similar function in the release of neurotransmitters from nerve cells. Because SLC10A4 is located in the mast cell granules and the storage of granule-associated mediators seemed intact in *Slc10a4* ^−/−^ BMMCs, it is possible that SLC10A4 influences the release of granule mediators by controlling the late events of mast cell degranulation. However, granule mediator release was intact after treatment with the Ca^2+^-ionophore in the *Slc10a4* ^−/−^ BMMCs, thus, SLC10A4’s function should then be controlled by signalling events triggered by receptor (FcεRI) activation. This warrants further studies of how the late signalling events differ between degranulation caused by activation via FcεRI and Ca^2+^-ionophore A12387 in mast cells.

Mast cells contain one of the largest depots of histamine found in mammalian cells^43^. Dopamine, like histamine, belongs to the monoamine neurotransmitter family and have similar requirements for their storage and uptake^44^. Interestingly, studies of SLC10A4 in the nervous system have demonstrated that SLC10A4 can regulate the release and reuptake of dopamine, with behavioural deficits resulting from impaired dopamine homeostasis^10^. *Slc10a4* ^−/−^ mice display increased sensitivity to amphetamine, and have lower brain levels of dopamine. The proposed mechanism behind the deficiencies in the central nervous system observed in *Slc10a4* ^−/−^ mice has been suggested to be a consequence of decreased vesicular monoamine re-uptake^10^. The lower uptake may be caused by reduced presence of vesicular counter ions in the synaptic terminals, such as ATP. Monoamines are positively charged at neutral pH. Therefore, to enter the more acidic vesicles, they need to overcome an electrical gradient in the form of protons^44^. Similarly, the acidic environment in mast cell granules poses a challenge for granular uptake of positively charged histamine via VMAT2. In mast cell granules, highly negatively charged heparin and chondroitin sulphate E proteoglycans serve to counterbalance positive charges^4^. These proteoglycans are also responsible for the staining of metachromatic mast cell granules, which occurs upon incubation with basic histological stains such as May-Grünwald/Giemsa. In our study, the *in vitro* differentiation of mast cells judged by the proportion of metachromatic granules was intact in the SLC10A4-deficient mast cells (Fig. 2E). This suggests that the storage of proteases and histamine, which depend on these negatively charged proteoglycans^4, 5, 45^, were intact. In addition, western blot and confocal microscopy demonstrated a similar content of the granule localized protease mMCP-6 in SLC10A4 deficient mast cells and their controls. However, for monoaminergic nerve cells, it has been proposed that ATP may act as a counter ion to alleviate an electrical gradient from the positively charged dopamine^10^. Although the proteoglycans likely play a role in counteracting positive charges in mast cells, it remains possible that ATP could participate in this process. Interestingly, the levels of ATP in the supernatant after IgE/antigen-mediated degranulation of SLC10A4 lacking mast cells was only one third of the levels detected in the supernatant from wild type mast cells. Live cell imaging of IgE/antigen-mediated degranulation process demonstrated that the fluorescent signals originating from ATP localised to the granules of *Slc10a4* ^−/−^ BMMCs were retained to a higher degree than in wild type BMMCs post-activation. These data suggest that *Slc10a4* ^−/−^ BMMCs degranulated less than their wild type counterparts. Therefore, we speculate that SLC10A4 either facilitates the transport of or directly transports ATP into the mast cell granules.

The reduction in the release of granule-associated mediators *in vitro* translated to an effect on mast cell-mediated reactions *in vivo*. The PCA reaction in *Slc10a4* ^−/−^ mice was significantly reduced, suggesting that the release of histamine from mast cells lacking SLC10A4 was impaired *in vivo*. Further, itching, classically triggered by injection of compound 48/80 that leads to release of histamine from mast cells, was affected. Injection of compound 48/80 caused significantly less scratching events in the *Slc10a4* ^−/−^ mice than in the wild type mice. Control and SLC10A4-deficient mice responded similarly to an injection of histamine demonstrating a normal reaction to histamine in the skin of *Slc10a4* ^−/−^ mice. Even though the number of scratch events was fewer in the *Slc10a4* ^−/−^ mice, the time spent on scratching during each of the events (bout time) was longer than those of the controls. Possibly, the histamine-mediated scratching events were occurring so rapidly that a new scratch-event tended to occur before the first one naturally ended, resulting in shorter and faster scratching-behaviour in wild type mice. Alternatively, the injection of 48/80 also stimulates release of another SLC10A4-regulated mediator, which has a blocking effect on the scratch reaction mediated by the nerves. This would also lead to a prolonged scratching behaviour in mice lacking SLC10A4. Altogether, the results from the PCA and itching experiments demonstrate that SLC10A4 regulates mast cell-related reactions *in vivo*.

In conclusion, our study for the first time identifies SLC10A4 as a regulator of mast cell degranulation. Our data imply that SLC10A4 affects the amount of granule-associated ATP upon mast cell activation with consequences for granule fusion with the plasma membrane, and therefore affects the efficiency of the release of granule-associated mast cell mediators. Thus, SLC10A4 is a potential novel drug target for mast cell-related diseases such as allergies.

## Methods

### Mice

SLC10A4 (*Slc10a4* ^−/−^) deficient mice were generated in 129/SvEvBrd mice by Texas A&M Institute^10^. These *Slc10a4* ^+/−^ were backcrossed to a C57BL/6 background for three generations and used in all experiments as sex-matched littermates, except for the passive cutaneous anaphylaxis experiments. For these experiments, the third generation of *Slc10a4* ^+/−^ mice on C57BL/6 background were backcrossed to a BALB/c strain originally obtained from Bommice (Ry, Denmark) for nine generations before use. The mice used for experiments were bred in-house and all the experiments were performed following the rules and regulations of the Swedish Board of Agriculture and the National Veterinary Institute. All experiments were approved by the Stockholm Norra animal experiments ethics committee, Stockholm animal experiments ethics committee or Uppsala animal experiments ethics committee.

### Culture of bone marrow-derived mast cells

The mice were euthanized by isoflurane overdose or neck dislocalisation. Bone marrow was harvested from femur and tibia by flushing the bones with RPMI-1640 complete medium (RPMI 1640 containing 100 U/ml penicillin, 100 μg/ml streptomycin, 10 μg/ml gentamicin, 2 mM L-glutamine, 0.1 mM nonessential amino acids, 10 mM HEPES, 50 μM 2-ME, 1 mM sodium pyruvate, and 10% heat-inactivated FCS (all from Sigma-Aldrich)). Harvested cells were spun at 417 × g for 5 min at room temperature and the pellet was resuspended in RPMI-1640 complete medium enriched with 50 ng/ml each of SCF (Peprotech Nordic, Stockholm, Sweden) and IL-3 from the X63 supernatant^46^. The number of viable cells was determined by trypan blue exclusion on a hemocytometer, adjusted to 5 × 10^5^ cells/ml, twice per week and cultured in humidified 37 °C incubator with 5% CO_2_. The percentage of FcεRI^+^ c-Kit^+^ mast cells were determined by flow cytometry on a LSR II (BD Biosciences, eBioscience, Hatfield, UK) using PE-Cy7 anti-c-Kit (2B8) and PE anti-FcεRI (MAR-1) antibodies (BD Biosciences, eBioscience, Hatfield, UK) after at least three weeks of culturing. The data was analysed using FlowJo (Tree Star Inc., Ashland, OR, USA). BMMC cultures containing 92–97% c-Kit^+^ FcεRI^+^ cells were used. In some experiments, the mast cell maturation was followed during *in vitro* culture. Samples from these cultures were taken in triplicates twice a week. The cells were cytospun onto glass slides (Shandon Cytospin 2) and were allowed to dry over night before staining by May-Grünwald/Giemsa (Sigma-Aldrich) using a standard protocol. The cells were imaged using a Nikon Eclipse Ni_U microscope, 400x magnifications. The software NSI-Elements BR 64-bit was used for capturing and editing, with automatic exposure time and medium contrast. All samples were scored blindly for presence or absence of fully matured granules within the cells during the developing period from the start of the culture to day 32 *in vitro*. A digital grid was applied to every picture and nine-squares/slide was analysed (at least 30 cells per slide were evaluated). The mean percentage of fully matured BMMCs was calculated from a total score per replicate/sample.

### Confocal Microscopy

Approximately 5 × 10^4^ BMMCs of each genotype were cytospun (Shandon Cytospin 2) at 500 rpm for six min to attach the cells onto glass slides. The cells were fixed with 4% paraformaldehyde for 10 min, followed by repeated washing with PBS (3 × 10 min). After blocking and permeabilization with 5% donkey serum and 0.1% Triton X-100 in PBS over night at 4 °C, rabbit anti-SLC10A4 1:1000 (HPA028835, Sigma-Aldrich) and rat anti-mMCP-6 1:1000 (MAB3736, R&D Systems) were added in blocking solution. After staining for 10–12 hours at 4 °C, the cells were washed repeatedly and the secondary antibodies anti-rabbit-Alexa Flour 488 and anti-rat-Alexa Flour 647 (Jackson ImmunoResearch Europe LTD, UK) and DAPI (for nuclear counterstaining) were added. After one hour incubation at room temperature, the cells were washed and mounted with Mowiol (Sigma-Aldrich). A Zeiss laser point scanning confocal microscope, LSM 510 Meta (Carl Zeiss, Jena, Germany) was used. Image processing was performed with the software LSM 5 (Zeiss, Germany). Control staining for background fluorescence was kept in blocking solution until secondary antibodies were added. Immunohistochemistry co-staining, for expression of SLC10A4 and mMCP-6 were analysed by z-stack imaging by IMARIS 7.4 (Bitplane AG, Zurich, Switzerland).

### ***In vitro*** stimulation assay

Mast cells were seeded at 1 × 10^6^ cells/ml and sensitized over night with anti-TNP IgE (prepared in-house from IgELb4^47^) at a final concentration of 2 µg/ml. The next day, cells were washed twice with PBS for removal of excessive IgE antibody and the cell pellet was resuspended in supplemented culture media (RPMI-1640 supplemented with 2 mM L-glutamine, 100 U/ml penicillin, 100 μg/ml streptomycin, 10 μg/ml gentamicin, 0.1 mM nonessential amino acids, 10 mM HEPES, 50 μM 2-ME, 1 mM sodium pyruvate, 20 g/l bovine serum albumin (A3912 BSA, Sigma) and 50 ng/ml of each SCF and IL-3). The cell concentration was adjusted to 1 × 10^6^ cells/ml before seeding to a 24-wells plate. For activation, the following reagents were added to obtain a final concentration of 100 ng/ml OVA-TNP, 2 µM Ca^2+^-ionophore (A23187) or vehicle (supplemented culture media). After one or 18–24 h activation (for IL-6 release), 600 µl cell suspension from each well were transferred to tubes and centrifuged for 500 × g for 5 min at 4 °C. The supernatant and cell fraction were analysed for presence of mediators either directly or after snap freezing and storage at −80 °C until analysis.

### Quantifications of mast cell mediators

For quantification of β-hexosaminidase, mast cell supernatants were collected directly after *in vitro* stimulation and cell pellets were lysed. To assay for retained granulated molecules, 500 µl 1.2% freshly prepared Triton-X100 was added and vortexed briefly. Sixty µl supernatant and cell lysate was added in triplicates to a 96-well ELISA plate and incubated for two hours at 37 °C with 60 µl of 4-nitrophenyl-N-acetyl-β-D-glucosaminide (N9376, Sigma) [3 mg/ml in 80 mM citric acid buffer]. The reaction was quenched by adding 120 µl of L-glycine [0.2 M] and the reaction analysed by absorbance measurement at 405 nM (Versamax microplate reader, Molecular Devices). Supernatants from activated mast cells were also analysed by the following kits: Histamine (BA E-5800; Labor Diagnostika Nord GmbH, Germany), Prostaglandin D2 (MBS262231; MyBioSource, San Diego, USA), ATP (Luciferin-Luciferase Bioluminescence assay (FL-AA, Sigma)) and IL-6 (CA92121, 88-7064-88; eBioscience Inc., San Diego, USA) according to the manufacturer guidelines. For tryptase activity measurements, 20 µl of fresh supernatant was diluted with 80 µl PBS and 20 µl of the substrate S-2288 (82083239, Chromogenix, NY) was added. Immediately thereafter, the absorbance was measured every 30 sec for 45 min at 405 nm and the Vmax expressed as mOD/min determined (Versamax microplate reader, Molecular Devices).

### Live cell imaging of mast cells upon IgE/antigen-induced mast cell activation

For analysis of the time course of mast cell degranulation, approximately 5 × 10^5^ cells were stained with 20 nM quinacrine (Sigma) for 10 min in humidified 37 °C incubator with 5% CO_2_. After washing twice with 37 °C pre-warmed PBS, cells were resuspended in 150 µl of supplemented media and seeded to a 35 mm glass bottom microwell dish (#P35GC-1.5-10-C, MatTek Corporation, MA, USA). BMMCs were either stimulated with 100 ng/ml of OVA-TNP to initiate degranulation or left untreated to evaluate impact of auto bleaching under the microscope. Degranulation was monitored by time-lapse on a confocal microscope (Zeiss LSM 510 Meta instrument; 63x magnification, pinhole 1.4 and 1.29 air unit). Z-stack images of 10 pictures/stack and 4 scans/slide were taken every 30 sec over a period of 75 min. Imaging analysis was done with IMARIS 7.4 (Bitplane AG, Zurich, Switzerland)

### Western blot

*Slc10a4* ^−/−^ and wild type BMMCs were lysed with RIPA buffer (150 mM NaCl, 1% NP-40, 0.5% Sodium deoxycholate, 0.1% SDS, 50 mM Tris, pH 8.0, all from Sigma) supplemented with complete protease inhibitor cocktail (Roche) and total protein concentrations determined. Cell lysates were stored at −20 °C until analysis. Twenty µg BMMC lysate was separated on 4–15% mini-PROTEAN^®^ TGX^™^ precast gel (Bio-Rad Laboratories AB, Sweden) and transferred to a nitrocellulose membrane (Bio-Rad) in a semi-dry electrophoretic transfer cell (Bio-Rad). The membrane was blocked with 2% BSA in TBST (50 mM Tris pH 8, 150 mM NaCl, 0.05% Tween® 20) and incubated with primary antibody in the same blocking solution for 16 h at 4 °C, followed by four washes in TBST. The membrane was then incubated with secondary antibody conjugated to horse radish peroxidase for 1 h at room temperature followed by washing in TBST. The protein bands were detected with ECL-prime luminol reagent (Pierce™, ThermoFisher Scientific) in a gel documentation system (Chemi-Doc, BioRad). Antibody dilutions were: rabbit-anti-SLC10A4, 1:2000 (HPA028835, Sigma), rat-anti-mMCP-6 1:2000 (MAB3736, R&D Systems) mouse-Actin beta 1:5000 (Cat: AC-15 Novus Biologicals, Colorado, USA), mouse-anti rabbit-HRP 1:10,000 (Cat: 211-032-171, Jackson lab), goat-anti rat-HRP 1:10,000 (Cat: NA935, GE Healthcare Life Sciences), goat-anti mouse-HRP 1:2500 (Cat: 62–6520 Invitrogen, Life Technologies). Quantitative analyses of the western blot results were obtained using ImageJ.

### Passive cutaneous anaphylaxis

Mice were anesthetized and injected intradermally in the left ear with 10 µl [1 mg/ml] anti-OVA IgE prepared in-house from the TOε hybridoma^48^ in PBS and 10 µl PBS in the right ear, as internal control. After 24 h, the sensitized mice were given 200 µl OVA containing 0.5% Evans Blue in PBS [0.25 µg/µl] intravenously in the tail and the animals were euthanized after 20 min and the ears removed for colorimetric analysis. The ears were weighed to calculate normalization, and the extravasation of Evans Blue was analyzed by addition of KOH [1 M] 37 °C on shake overnight and neutralized by adding 0.2 M H_3_PO_4_:acetone (5:13, v/v). The digested ear solution was spun twice at 16000 rcf for 10 min to remove cellular and tissue debris. Absorbance was measured in triplicate samples at 620 nm and normalized against tissue weight.

### Itch assay

All behavioural tests were performed on adult (>7 weeks old) male *Slc10a4* ^−/−^ mice and their wild type littermates in a controlled environment of 20–24 °C, 45–65% humidity and 12 hours day/night cycle. The observers were blind to the genotype. The mice were injected intradermally in the neck with 50 µl 48/80 or histamine [2 µg/µl] in saline (9% physiological NaCl) or as basal control animals were injected with 50 µl of saline as vehicle. One hour video recordings were used to quantify scratch behaviour, and were later scored blindly for analysis of individual behaviour patterns.

### Statistical analysis

The software GraphPad 5 was used for statistical analysis. Significance definitions were set as follows, *P* < 0.05, 0.01 or 0.001 and are represented by *^,^ ** and *** Respectively.

## Electronic supplementary material

Supplementary material
