# Supplementary material for: SLC10A4 regulates IgE-mediated mast cell degranulation in vitro and mast cell-mediated reactions in vivo
Source: Sci Rep. 2017 Apr 24;7:1085. doi: 10.1038/s41598-017-01121-8 (PMC5430724; doi:10.1038/s41598-017-01121-8)
Supplement: Supplementary file 1 — Supplementary material [file 41598_2017_1121_MOESM1_ESM.doc]

**SLC10A4 regulates IgE-mediated mast cell degranulation in vitro and mast cell-mediated reactions in vivo**

**Hanna Pettersson**^1*^**, Behdad Zarnegar**^2^**, Annika Westin**^2^**, Viktor Persson**^2^**, Christiane Peuckert**^1^**, Jörgen Jonsson**^1^**, Jenny Hallgren**^2+^ **and Klas Kullander**^1+^

^1^ Department of Neuroscience, Uppsala University, Box 593, 751 24 Uppsala, Sweden

^2^ Department of Medical Biochemistry and Microbiology, Uppsala University, Box 582, 751 23 Uppsala, Sweden

* Current address: Department of Organismal Biology, Uppsala University, Norbyv. 18A, 752 36 Uppsala, Sweden

^+^ Correspondence: [klas.kullander@neuro.uu.se](mailto:klas.kullander@neuro.uu.se) (KK), [jenny.hallgren@imbim.uu.se](mailto:jenny.hallgren@imbim.uu.se) (JH),

shared last authors

**Supplementary Figures**

**
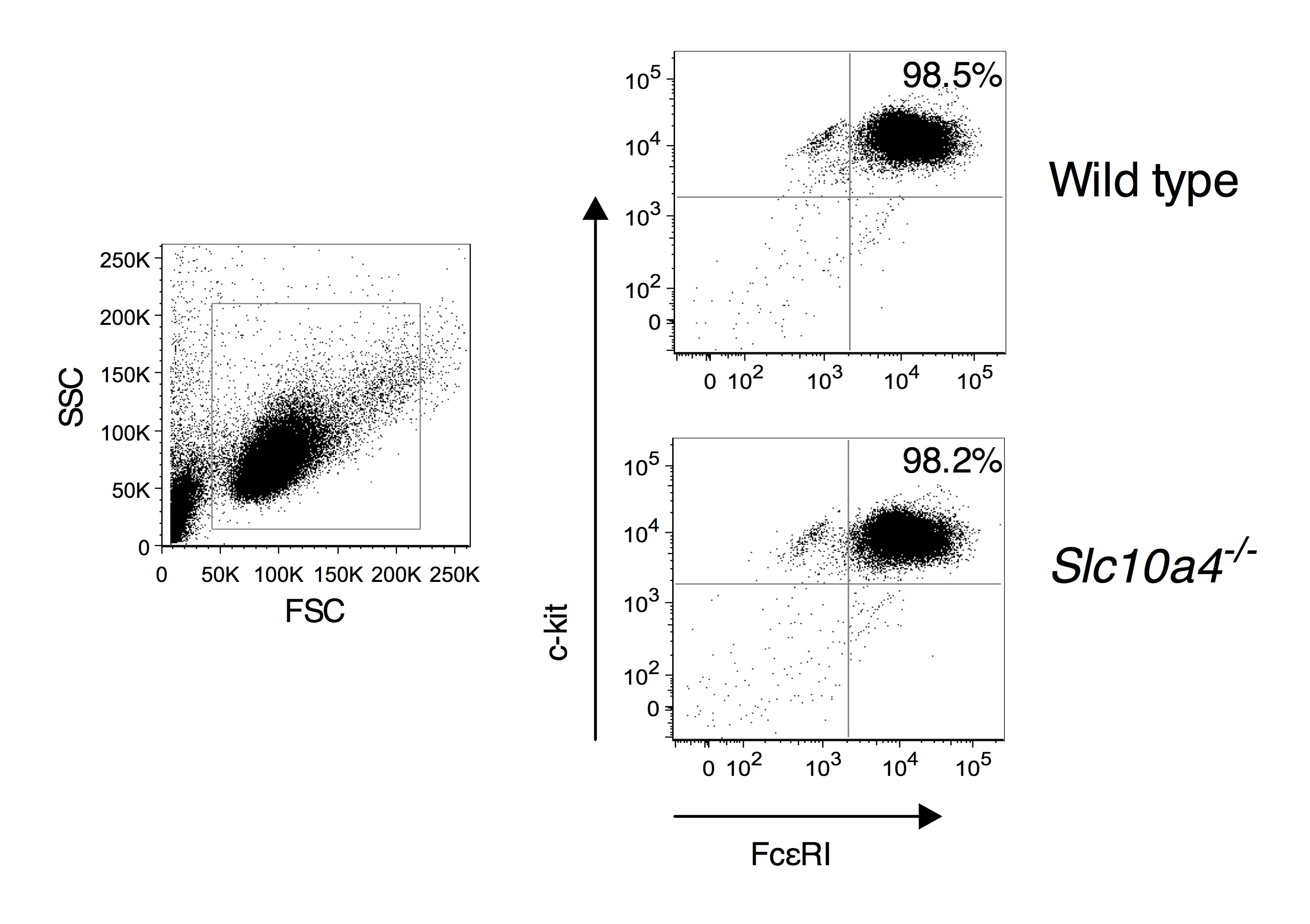
**

**Supplementary Figure S1. A similar percentage of c-kit^+^ FcεRI^+^ mast cells were obtained in BMMC-cultures of wild type and *Slc10a4^-/-^*bone-marrow cells.**

Bone marrow cells were cultured in the presence of IL-3 and SCF. The percentage of c-kit^+^ FcεRI^+^ BMMCs was assessed by flow cytometry routinely before any experimentation. The graph displays a representative flow cytometry dot plot.


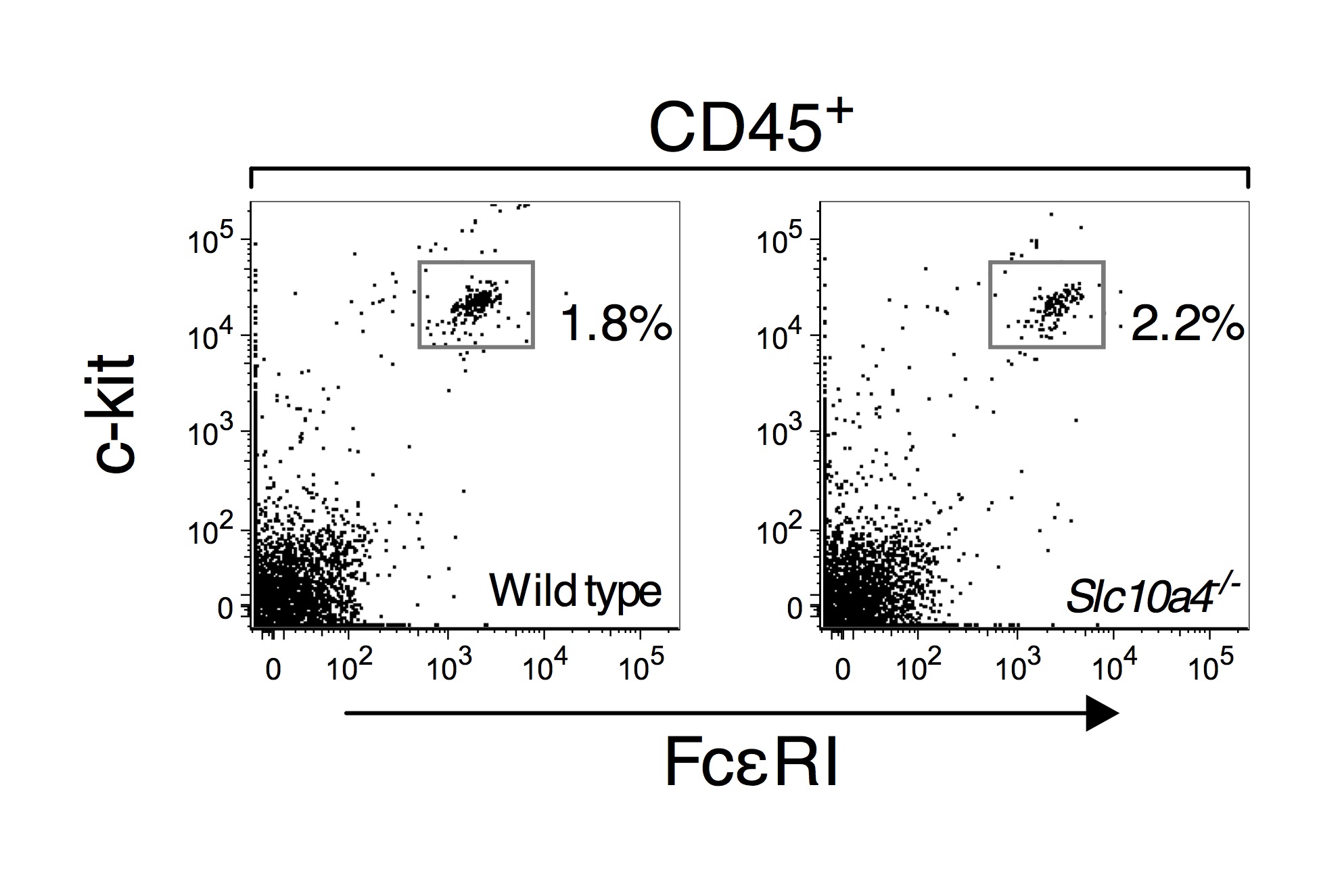


**Supplementary Figure S2. Wild type and *Slc10a4^-/-^* mice have a similar percentage of peritoneal mast cells.**

Representative flow cytometry dot plots of CD45^+^ c-kit^+^ FcεRI^+^ peritoneal mast cells.


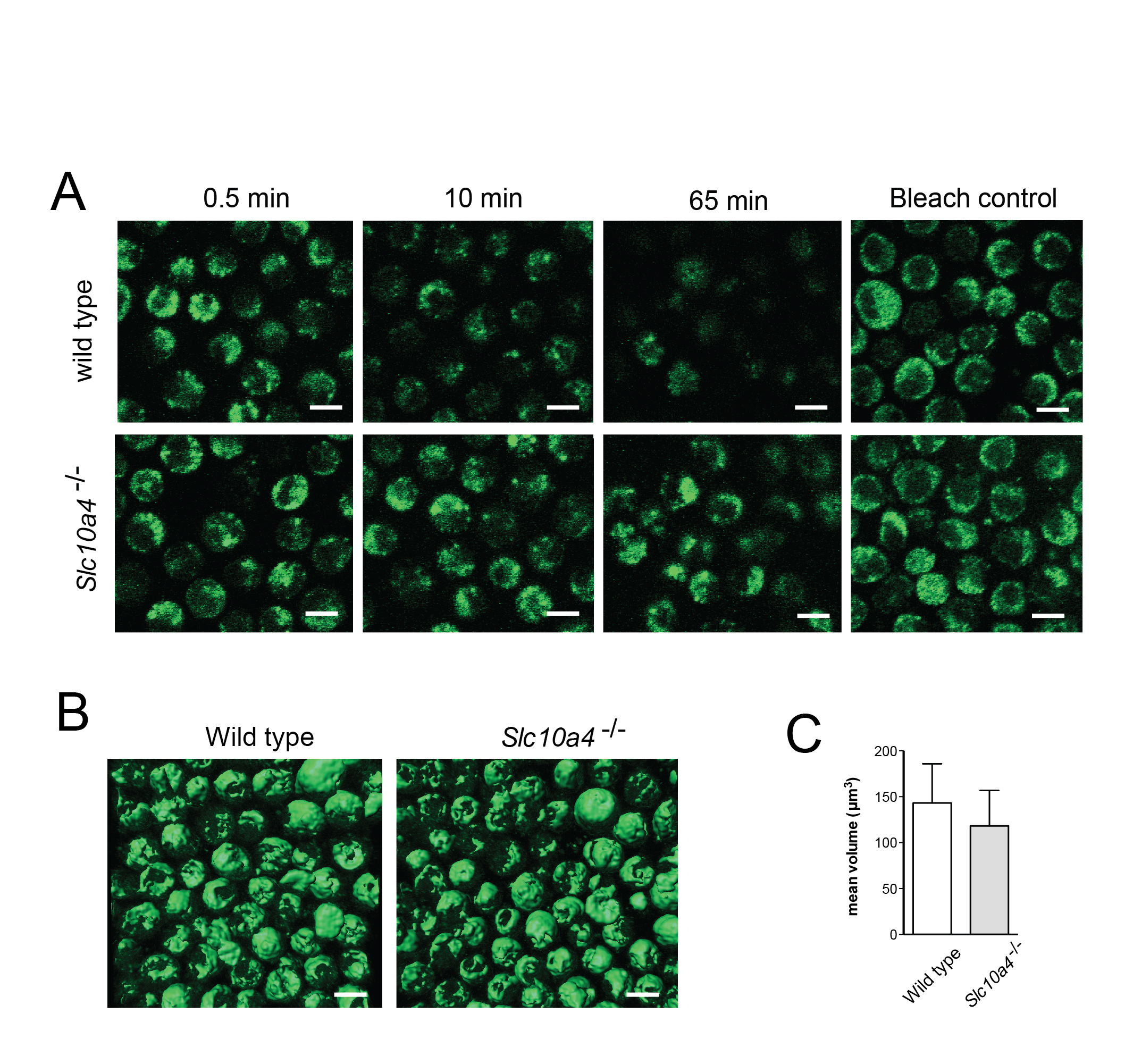


**Supplementary Figure S3. Sensitized wild type and *Slc10a4*^-/-^ BMMCs have similar intracellular ATP levels before activation.**

(**A**) Anti-TNP IgE-sensitized wild type and *Slc10a4^-/-^* BMMCs were stained with the ATP-binding drug quinacrine (green) and treated with OVA-TNP to trigger degranulation. The reactions were monitored over 75 min. As a control, untreated BMMCs were left in the confocal microscope for monitoring auto-bleach decrease in staining due to the laser exposure for the same time span (Bleach control). (**B**) Sensitized *Slc10a4^-/-^* BMMCs were stained with the ATP-binding drug quinacrine (green), left untreated and visualized by live cell imaging in a confocal microscope and converted to volumes using Imaris software. (**C**) Quantification of fluorescent volumes in (B). Bar = 10 µm.
